# Regenerative potential of MSC-derived secretomes in radiation-induced xerostomia: Role of SDF-1, IL-10, and VEGF

**DOI:** 10.1016/j.jobcr.2026.101453

**Published:** 2026-04-16

**Authors:** Sri Wigati Mardi Mulyani, Nastiti Faradilla Ramadhani, Deny Saputra, Yunita Savitri, Putri Alfa Meirani Laksanti, Tengku NEBTA. Noor

**Affiliations:** a Department of Dentomaxillofacial Radiology, Faculty of Dental Medicine, Universitas Airlangga, Prof. Dr. Moestopo Street No. 47, Surabaya, 60132, Indonesia; b Master of Dental Health Science Study Program, Faculty of Dental Medicine, Universitas Airlangga, Prof. Dr. Moestopo Street No. 47, Surabaya, 60132, Indonesia; c Malaysian Armed Forces Dental Officer, 609, Armed Forces Dental Clinic, Kuching, Serawak, Malaysia

**Keywords:** Secretome MSCs, IL-10, Ionizing radiation, Regenerative therapy, SDF-1, VEGF

## Abstract

**Purpose:**

This study aims to analyze the potential of MSC-derived secretomes, specifically SDF-1, IL-10, and VEGF, as an alternative therapy for xerostomia or other disorders caused by ionizing radiation.

**Materials and methods:**

This research is an experimental laboratory-based analytical study. The samples were secretomes derived from mesenchymal stem cells (MSCs) isolated from human umbilical cords. The MSCs were divided into two groups: the control group (K), which consisted of MSC cultures without X-ray exposure, and the treatment group (P), consisting of MSC cultures exposed to X-rays. Each cell culture was incubated until day 3 and day 7. The secretion levels of SDF-1, IL-10, and VEGF were then measured using a sandwich ELISA test.

**Results:**

The study showed that the average secretion levels of SDF-1, IL-10, and VEGF increased significantly after exposure to 0.16 mSv ionizing radiation, both on day 3 and day 7, compared to the control group without radiation exposure. However, the average secretion levels of SDF-1, IL-10, and VEGF in the treatment group on day 7 (P.7) were lower than those on day 3 (P.3).

**Conclusion:**

Secretomes from MSC cultures have great potential as an alternative therapy for xerostomia or other disorders caused by ionizing radiation, serving as a cell-free therapy. Secretomes contain various bioactive molecules such as cytokines, chemokines, antioxidants, and growth factors that play a crucial role in cell and tissue regeneration.

## 1 Introduction

Radiotherapy is a widely used alternative therapy for cancer patients in the head and neck region, but it is associated with unavoidable side effects. One of the most common and inevitable side effects of radiotherapy in this area is salivary gland dysfunction, which can progress into dry mouth syndrome or xerostomia.^1^ Xerostomia is the subjective sensation of dry mouth, which may be related to reduced or absent saliva secretion. Generally, the dryness of the mouth becomes noticeable when salivary flow is reduced by more than 50%.^2^ Xerostomia significantly affects quality of life, causing bad breath, sore throat, tongue inflammation, loss of taste, gingivitis, and other discomforts.^3^ Radiotherapy has been shown to alter the oral environment, including salivary flow, pH balance, immune response, and oral microbiota composition, which may contribute to periodontal tissue damage and increased susceptibility to oral diseases. Additionally, hyposalivation and reduced buffering capacity of saliva following radiation exposure further exacerbate tissue injury and functional impairment of salivary glands.^4^

There are no effective regenerative therapies for treating this condition. Current clinical treatments are limited to palliative care. However, a promising new regenerative therapy using mesenchymal stem cells (MSCs) offers an alternative approach for xerostomia treatment.^5^ MSCs have become the focus of research in various fields, including tissue regeneration, cell therapy, and the treatment of inflammatory diseases. Their ability to modulate immune responses and produce growth factors that accelerate tissue repair makes them a promising candidate for addressing various medical conditions.^6^

Mesenchymal stem cells are multipotent stem cells have capability of differentiating into various cell types, such as chondrocytes, adipocytes, osteoblasts, and salivary gland epithelial cells. MSCs have been studied extensively in both in vitro and in vivo experimental studies, as well as in clinical trials, for a variety of conditions. Their unique properties, including homing ability, anti-inflammatory effects, low immunogenicity, and potential to repair damaged tissues, have made them a promising area of study for regenerative medicine.^7^

Currently, much of the focus in stem cell therapy is on stem cell transplantation. However, challenges such as weak engraftment and limited survival of transplanted cells remain significant limitations. Additionally, complexities in delivering stem cells effectively into the body and determining optimal dosages in clinical trials are issues that require further study. These challenges highlight the need for more effective approaches to deliver the functions and potential of stem cells in preventing and regenerating damage to salivary gland cells caused by ionizing radiation. A more targeted approach is required to address specific biological mechanisms involved in salivary gland injury, including cell recruitment, inflammation control, and vascular regeneration.^8^

Small molecules secreted by MSCs, known as metabolites or secretomes, have gained attention as a potential cell-free therapy for tissue regeneration, offering advantages over cell-based therapies using MSCs. Research has shown that MSCs secrete metabolites containing various cytokines, chemokines, growth factors, proteins, and antioxidants, such as glutathione, stromal-derived factor-1 (SDF-1), vascular endothelial growth factor (VEGF), and interleukin-10 (IL-10), during early subculture. These secreted factors significantly enhance MSCs' regenerative capabilities.^9^

SDF-1 is a chemokine that plays a role in cell migration/homing, growth, and regeneration in damaged areas. More specifically, SDF-1 functions as a homing signal that recruits endogenous stem cells to migrate toward injured salivary gland tissue, thereby enhancing intrinsic repair mechanisms.^10^ VEGF acts as an anti-apoptotic factor and promotes angiogenesis, supporting regeneration by stimulating neovascularization and enhancing wound healing through vascular and trophic (pro-angiogenic) effects.^11^ While IL-10 serves as an anti-inflammatory agent with significant anti-fibrotic properties, reducing inflammatory responses and preventing further tissue damage following radiation exposure.^12^ Previous studies suggest that the regenerative effects of stem cells are largely attributed to their paracrine activity. Therefore, this study focuses on using MSC metabolites or secretomes as the therapeutic agent. Based on these considerations, we aim to develop a cell-free therapy utilizing the potential of MSC-derived secretomes as an alternative treatment for xerostomia by analyzing the secretion levels of SDF-1, IL-10, and VEGF.

## 2 Materials and Methods

In this study, secretomes were isolated from mesenchymal stem cells (MSCs) derived from human umbilical cords (human umbilical cord-derived mesenchymal stem cells). This study was conducted following ethical approval from the Airlangga University Faculty of Dental Medicine Health Research Ethical Clearance Commission (No: 0814/HRECC.FODM/VIII/2024). A total of 1 × 10^51^ times 10^51^ × 10^5^ MSCs were seeded onto 100-mm culture dishes and incubated at 37 °C until they reached confluency. At the fourth passage, hUC-MSCs were collected and characterized to confirm their identity as mesenchymal stem cells. Characterization was performed using flow cytometry to assess specific markers, including CD105, CD90, CD45, and CD34.

The MSCs were divided into two groups: Control group (CG) MSCs cultures without x-ray exposure and Treatment group (T) MSCs culture exposed to x-rays. Each experimental condition was performed in 12 independent batches (biological replicates) to ensure reproducibility of the secretome production. Cell cultures both were incubated until days 3 and days 7. X-ray irradiation was performed using a Belmont Phot-X S 505 DC Dental X-Ray device (70 kV, 7 mA). The treatment group received eight exposures, with a cumulative dose of 0.20 mSv. Each exposure was delivered for 0.15 s. The irradiation procedure was conducted in a dedicated X-ray room to ensure standardized exposure conditions.

On days 3 and 7, the metabolites were collected from the cultures. The total volume of conditioned medium (secretome) collected from each 100-mm dish was approximately 24 ml, and the collected secretome was stored at −80 °C until further analysis. For each experimental assay, 2 ml of secretome was used per sample/flask. The collected metabolites were ultrafiltered using a membrane with a molecular weight cutoff of 3 kDa to remove small molecular components such as salts and amino acids. The filtration was carried out using Amicon Ultra-15 centrifugal filter-units.

The secretion levels of stromal-derived factor-1 (SDF-1), interleukin-10 (IL-10), and vascular endothelial growth factor (VEGF) were analyzed using a sandwich ELISA (Catalog No. MDB0052H). Secretomes were collected from the cultures and tested for the release of selected cytokine, chemokine, and growth factor panels. A four-parameter logistic curve was plotted for the standards, and the results for the test samples were calculated accordingly.

## 3 Statistical analysis

The results of this study will be analyzed and presented in the form of data tabulation and bar charts. Research data were analyzed using the Statistical Package for the Social Science software (SPSS). Next, statistical testing was carried out on the data obtained by testing normality using the Kolmogorov-Smirnov test and homogeneity using the Levene test. Differences in variables will be tested using the one-way ANOVA test and followed by the Post-Hoc Tukey HSD test.

## 4 Results

This research was conducted to analyze the potential of Mesenchymal Stem Cells (MSCs) secretomes as an alternative therapy material for xerostomia caused by ionizing radiation through the levels of secretion of SDF-1, IL-10 and VEGF in the MSCs cultures presented. against X-ray radiation. The research results were analyzed using the sandwich ELISA test by looking at the levels of SDF-1, IL-10 and VEGF secreted from the secretome of MSCs as seen in Table 1, Table 2, Table 3, Table 4, Table 5, Table 6.

**Table 1:** ELISA analysis of SDF-1.

	Group	SDF-1			
		3rd Day		7th Day	
**Control Group**	**1**	0,222	0,217	0,105	0,129
	**2**	0,232		0,118	
	**3**	0,211		0,148	
	**4**	0,204		0,143	
**Treatment Group**	**1**	0,326	0,287	0,198	0,184
	**2**	0,262		0,19	
	**3**	0,247		0,168	
	**4**	0,312		0,179

**Table 2:** Post-Hoc analysis of SDF-1 expression.

Groups	K.3	K.7	P.3	P.7
**K.3**		0,003∗	0,093	
**K.7**				0,021∗
**P.3**				0,028∗
**P.7**				

∗Significance p < 0.05.

**Table 3:** ELISA analysis of IL – 10.

	Group	IL-10			
		3rd Day		7th Day	
**Control Group**	**1**	12,987	8849	6040	4410
	**2**	8939		5840	
	**3**	7529		1040	
	**4**	5941		4720	
**Treatment Group**	**1**	13,160	13,913	12,001	11,759
	**2**	15,302		11,671	
	**3**	14,031		10,722	
	**4**	13,158		12,640

**Table 4:** Post-Hoc analysis of IL-10 expression.

Groups	K.3	K.7	P.3	P.7
**K.3**		0,038∗	0,018∗	
**K.7**				0,001∗
**P.3**				0,459
**P.7**				

∗Significance p < 0.05.

**Table 5:** ELISA analysis of VEGF.

	Group	IL-10			
		3rd Day		7th Day	
**Control Group**	**1**	488,672	702,018	502,500	406,031
	**2**	897,073		460,625	
	**3**	778,144		241,786	
	**4**	644,183		419,214	
**Treatment Group**	**1**	1253,250	1316,749	770,266	758,569
	**2**	1433,947		754,364	
	**3**	1326,737		708,576	
	**4**	1253,061		801,071

**Table 6:** Post-Hoc analysis of VEGF expression.

Groups	K.3	K.7	P.3	P.7
**K.3**		0,015∗	0,000∗	
**K.7**				0,005∗
**P.3**				0,000∗
**P.7**				

∗Significance p < 0.05.

**Fig. 1:**
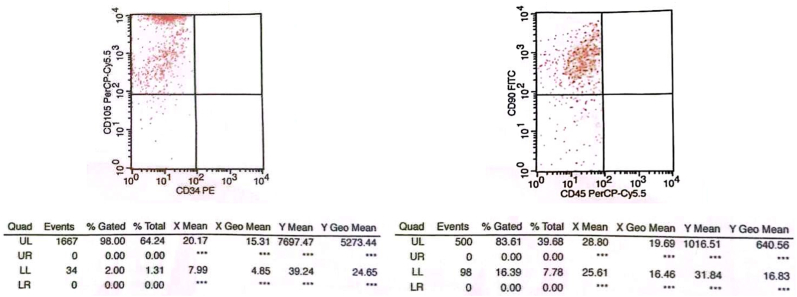
Flow cytometry results of umbilial-cord mesenchymal stem cells characterization.

Before being exposed to X-ray radiation, MSCs were first characterized by specific MSC marker proteins using flow cytometry to see the expression of CD105, CD90, CD45 and CD34 (Fig. 1) (see Table 4). Flow cytometry results on MSCs culture showed positive expression of CD105 and CD90 in 64,24% and 39.68%, respectively. Meanwhile, it showed negative expression of CD34 and CD45.

**Fig. 2:**
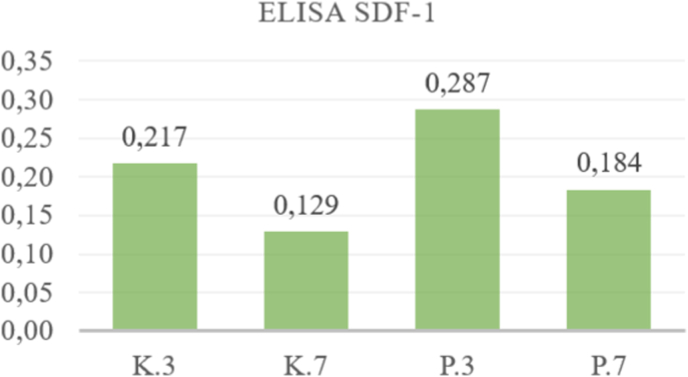
Mean of ELISA analysis SDF-1 3rd and 7th day.

**Fig. 3:**
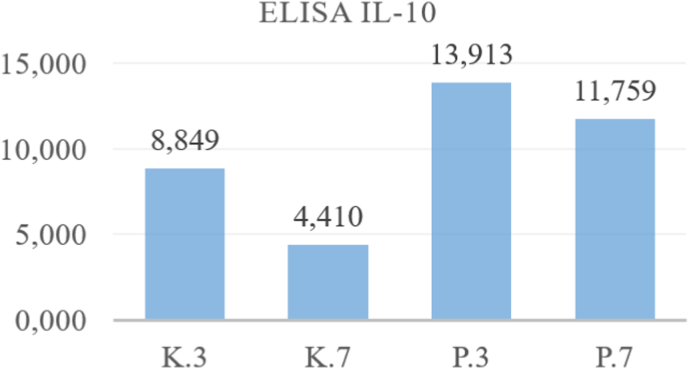
Mean of ELISA analysis IL-10 3rd and 7th day.

**Fig. 4:**
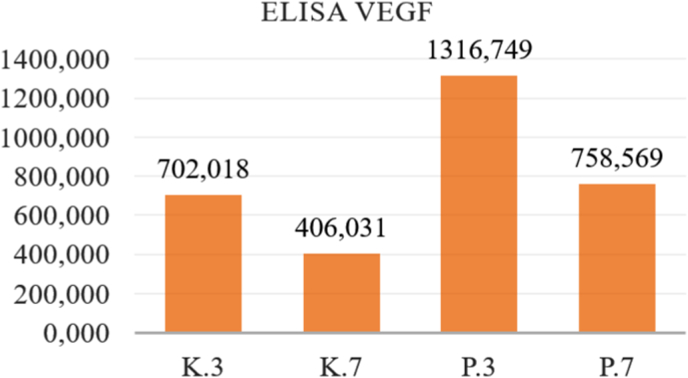
Mean of ELISA analysis VEGF 3rd and 7th day.

The results of the study were divided into four groups, namely the control group (K) without exposure to X-ray radiation and the treatment group (P) which was given X-ray exposure, then observations were carried out on the 3rd and 7th day as seen in Fig. 2, Fig. 3, Fig. 4.

## 5 Discussion

This research was conducted to test the potential of secretomes from Mesenchymal Stem cells (MSCs) as an alternative cell-based therapy material for Xerostomia therapy caused by radiation therapy through the levels of secretion of SDF-1, IL-10, and VEGF. Secretomes were collected from metabolites of cultured MSCs originating from the placenta or human umbilical cord (hUC-MSCs) that were exposed to ionizing radiation.

In this study, human umbilical cord (hUC-MSCs) was used as a source of mesenchymal stem cells because hUC-MSCs have immunomodulatory and anti-inflammatory properties. In addition, hUC-MSCs in vitro culture do not require a long time due to their higher self-renewal ability compared to mesenchymal stem cells isolated from other sources. Secretion of hUC-MSCs can also increase the migration of Endothelial Progenitor Cells (EPC), one of the important cell populations in angiogenesis.^13^

MSCs have been the focus of research in various fields, including tissue regeneration, cell therapy, and the treatment of inflammatory diseases. The ability of MSCs to modulate immune responses and secrete various cytokines, chemokines and growth factors that can accelerate tissue healing has made them a promising study material in the treatment of various medical conditions.^6^ Therefore, regenerative therapy with metabolites or secretomes from Mesenchymal stem cells (MSCs) is a cell-free based regenerative therapy that is quite promising for salivary gland disorders or xerostomia.

The results of the study showed that the mean levels of secretion of SDF-1, IL-10 and VEGF increased significantly after exposure to ionizing radiation of 0.16 mSv, both on observation This shows that radiation exposure to MSCs culture cells has caused an inflammatory reaction due to free radicals produced due to exposure to x-ray radiation so that in both treatment groups (P.3 and P.7) there was an increase in the secretion of IL-10 as an anti-inflammatory cytokine. Free radicals produced as a result of x-ray radiation can cause oxidative stress, triggering an inflammatory reaction that causes cell damage and results in cell death. This research also shows that radiation exposure can cause injury, thereby inducing MSCs to secrete SDF-1 and VEGF, which is a chemokine that plays a role in the process of migration/homing, cell growth and regeneration of damaged tissue. Meanwhile, VEGF is a growth factor that acts as an anti-apoptosis and angiogenesis process. Stem cells can modulate their secretome production in response to stress, injury, or environmental changes to prevent cell damage.

The efficacy of MSCs in regenerative therapy is not only limited to their ability to differentiate into various cell types, but also to their ability to modulate the surrounding cellular environment, namely by secreting a number of cytokines, chemokines and growth factors, such as vascular endothelial growth factor (VEGF), basic fibroblast growth factor (bFGF), interleukin-10 (IL-10), and stromal cell-derived factor-1 (SDF-1), among others. These results are in accordance with the results of several studies which show that MSCs can provide therapeutic effects through paracrine mechanisms, namely the release of growth factors and cytokines which can modulate immune responses, increase angiogenesis, and reduce inflammation.^14^ Many studies show that MSCs produce large amounts of secretomes. Which is larger than other cells. These secreted secretomes mediate many regenerative properties previously thought to belong to stem cells.^15^ Secretomes contain various protein compounds, growth factors, cytokines, and other signaling molecules. The secretome plays an important role in intercellular communication and regulation of the microenvironment around stem cells. They can influence proliferation, differentiation, and interactions with surrounding cells.

The results of the sandwich ELISA test in this study also showed that the mean secretion of SDF-1, IL-10 and VEGF in the treatment group on day 7 (P.7) decreased compared to day 3 (P.3). This is possibly because on day 3 monocytes begin to differentiate from pro-inflammatory M1 into anti-inflammatory macrophages (M2) which secrete anti-inflammatory cytokines, one of which is IL-10, which functions to inhibit the release of pro-inflammatory cytokines. On day 7, the final inflammatory phase, the transition to proliferation, has taken place so that the number of anti-inflammatory cytokines becomes more dominant as it moves towards the healing stage.^9^

In addition, secretome production by stem cells can be influenced by several factors, such as environmental conditions, stem cell differentiation status, and external signals, in this case the culture cell environment for example. Too long cell culturing time is one of the factors that can cause a decrease in the potential of MSCs, including the ability to secrete various bioactive chemokine compounds, cytokines and growth factors, including SDF-1, IL-10, and VEGF.

## 6 Conclusion

The secretome from MSCs has great potential as an alternative therapy for xerostomia or other disorders caused by ionizing radiation as a cell-free therapy. Secretomes contain various bioactive molecules such as cytokines, chemokines, antioxidants and growth factors which play an important role in cell and tissue regeneration.

## Patient's/guardian's consent

Not applicable.

## Ethical clearance

This study has received ethical approval from the Ethics Committee of Faculty of Dental Medicine, Universitas Airlangga, with the ethical clearance number 0814/HRECC.FODM/VII/2024 on August 1st 2024.

## Source of funding

This study received Airlangga Research Fund Grant with Grant Number 1637/B/UN3. LPPM/10.13039/100023865PT.01.03/2024.

## Declaration of competing interest

The authors declare that they have no known competing financial interests or personal relationships that could have appeared to influence the work reported in this paper.
